# The Role of Phosphate Fertilization on Physiological Responses of the Young *Bertholletia excelsa* Plants Grown in a P-Deficient Amazon Ferralsol

**DOI:** 10.3390/plants11212955

**Published:** 2022-11-02

**Authors:** Viviane Corrêa, José Gonçalves, Karen Costa, Igor Oliveira, José Santos, Sabrina Oliveira, Marciel Ferreira, Roberval Lima, Wagner Araújo, Adriano Nunes-Nesi

**Affiliations:** 1 Federal Institute of Education, Science and Technology (IFRO), Rua Rio Amazonas, 151, Jardim dos Migrantes, Ji-Paraná 76900-310, RO, Brazil; 2 Laboratory of Plant Physiology and Biochemistry, National Institute for Amazonian Research (MCTI-INPA), Avenida André Araújo, 2936, Aleixo, Manaus 69011-970, AM, Brazil; 3 Faculty of Agricultural Sciences, Institute of Studies in Agrarian and Regional Development (IEDAR), Federal University of South and Southeast of Pará (UNIFESSPA), Rodovia BR-230 (Transamazônica), Cidade Jardim, Marabá 68500-000, PA, Brazil; 4 Bionorte Graduate Program (BIONORTE), Amazonas State University, Rua Carvalho Leal A, 1777, Bairro Cachoeirinha, Manaus 69065-001, AM, Brazil; 5 Faculty of Agricultural Sciences, Federal University of Amazonas (UFAM), Avenida General Rodrigo Octavio Jordão Ramos, 1200, Coroado I, Manaus 69067-005, AM, Brazil; 6 Embrapa Western Amazon, Research and Development, Rodovia AM 010, km 29, Manaus 69010-970, AM, Brazil; 7 Departamento de Biologia Vegetal, Universidade Federal de Viçosa (UFV), Avenida PH Rolfs, s/n, Viçosa 36570-900, MG, Brazil

**Keywords:** ecophysiology, functional traits, nutrient use efficiency, plant nutrition, plasticity

## Abstract

Phosphorus (P) reacts with soil minerals, which makes it less available to plants. Considering that Amazonian soils have a low pH and nutrient availability, both of these properties contribute to an increase in P limitation. Here, we investigate how the addition of P to the substrate affects morpho-physiological traits of Brazil nut trees (*Bertholletia excelsa* Bonpl.). The experiment was carried out in a greenhouse with 24-month-old saplings, and the P treatments consisted of a control (Ferrasol without P addition) and 100, 200, 400, and 500 mg P kg^−1^ of added to the soil. When *B. excelsa* saplings were fertilized with phosphate, the N:P leaf ratio reduced from 50 to 26. Addition of P favored the photochemical efficiency of PSII (F_V_/F_M_), and the application of 200 mg kg^−1^ increased photosynthesis (*P_N_*) by 50%. Furthermore, phosphorus enhanced light and nutrient use efficiency. An increase in *B. excelsa* dry biomass was observed when 200 mg P kg^−1^ was added, with maximum yield occurring at 306.2 mg P kg^−1^. Physiological parameters suggest robust responses by *B. excelsa* to P fertilization. In addition, our findings reveal the critical role of P on *B. excelsa* growth in Ferralsol, as well as the potential of P fertilization to improve functional traits of this important Amazonian tree.

## 1. Introduction

Ferralsol is the main type of soil in the Amazonian environment and is characterized by high acidity and low nutrient concentrations [1,2]. Despite the low fertility, the Amazon tree species do grow in Ferralsol and benefit from nutrient-cycling mechanisms [3]. Nevertheless, the soil conditions for forestry plantations in degraded areas of the Amazon are extremely hostile.

In general, phosphorus (P) is the most limiting nutrient in amazon Ferralsols [4,5]. In this soil, naturally low phosphorus levels and soil adsorption have been identified as the main processes controlling P availability. [6]. Not coincidentally, soil availability of P has been used to explain variations and limitations in forest biomass and growth across the Amazon basin [7,8,9]. However, the question of whether plants will fulfill the P demand for an increase in biomass under elevated CO_2_ conditions remains to be answered [10]. Plant responses to these conditions encourage the use of P fertilization in order to increase tree growth, especially in degraded areas of the Amazon region, thus stimulating initiatives for the installation and management of forest plantations. Understanding the effects of low P availability and its supplementation in Amazon soils and in native tree species with potential for forest plantations, such as *B. excelsa*, is essential for silvicultural applications. These new possibilities, not yet confirmed by conventional plantations, offer an opportunity to improve silviculture in the Amazon [11,12,13].

The effects of P availability on plant growth and morpho-physiological traits depend on the P requirement of different plant species. Recent studies have demonstrated that some tree species exhibit different mechanisms that may allow them to survive and grow well even when there is low P availability [5,10,14,15]. These mechanisms involve changes in P fractionation, altered leaf and root morphological traits, as well as changes in biomass allocation, and phosphatase release. *Bertholletia excelsa* Bonpl., for instance, is planted in the Amazon, and rarely in these plantations is there any soil treatment. Even under these conditions of low P availability, this species achieves greater growth when compared to other native species [16,17]. Interestingly, *B. excelsa* can reach growth rates similar to those observed in exotic species, for example, eucalyptus, when planted in Amazonia conditions. [17,18]. It is noteworthy that the growth performance of *B. excelsa* can be considered superior when compared to other native or even exotic tree species cultivated under harsh soil conditions [13].

Recent studies have shown that *B. excelsa* exhibits high functional plasticity in response to drought stress and irradiance [12,14,15]. This functional trait makes it capable of exhibiting high physiological performance under controlled conditions (greenhouse), in native forests, or in different planting sites [12,13,16,19,20,21,22,23]. However, the interactions related to the physical, chemical, and biological factors of the site cultivation and silvicultural practices are not fully understood for *B. excelsa* plantations. In adult *B. excelsa* trees subjected to thinning, liming, and P fertilization, it was observed that thinning reduced photochemical efficiency, but on the other hand, liming and phosphate fertilization alleviated the effects of high irradiance stress and accelerated tree recovery [12]. 

These responses are of paramount importance since *B. excelsa* is one of the symbolic species of the Amazon region. This species is responsible for important environmental services and it is also the most widely used in extractive activities of non-timber forest products in the Amazon region or as a planted species in agroforestry systems due to the generation of income and food security [24,25]. Therefore, by displaying a powerful social appeal and with the high physiological plasticity of the *B. excelsa*, the improvement of the functional performance of this species is highly desirable for establishing productive tree plantations in the Amazon [13].

In light of the recent knowledge of *B. excelsa*’s plasticity, especially in terms of photosynthesis, it could be affirmed that this species shows high photosynthetic performance [11,12,20,21,22]. Fertilization improves the photosynthetic process of young *B. excelsa* plants in a degraded area in the central Amazon [23]. In addition, higher P availability in the soil is associated with higher fruit yield by *B. excelsa* trees [26]. However, there is still a lack of information regarding *B. excelsa’s* responses to P fertilization regarding the mechanisms that improve photosynthetic performance and plant growth in P-deficient Amazon Ferralsol. We hypothesize that greater photosynthesis in *B. excelsa* saplings, fertilized with P, is explained by high photosynthetic light use efficiency, which then leads to high growth rates. Thus, this study aimed to investigate the effects of increased P levels on morpho-physiological traits and plant growth of Brazil nut saplings. In addition, the improvements in photochemical performance and higher stomatal conductance in *B. excelsa* leaves in response to P in a Ferralsol typical of the Amazon are discussed.

## 2. Results

**Figure 1:**
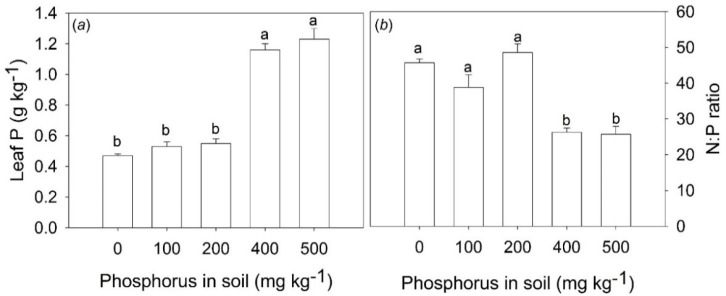
Leaf phosphorus content (**a**) and leaf N:P ratio (**b**) of *Bertholletia excelsa* saplings under increasing doses of phosphorus in a P-deficient Amazon Ferralsol. Bars are the means and lines above bars are the standard error. Different letters in the bars indicate significant differences between the means by the Tukey test at a 0.05 probability level.

The P soil fertilization increased leaf P content and decreased the N:P ratio only in treatments with the addition of 400 and 500 mg P kg^−1^ in the soil (Figure 1a,b). The P content in the leaves of these treatments was 140% higher when compared with control and treatments with the addition of 100 and 200 mg P kg^−1^ (Figure 1a). The N:P ratio in these treatments decreased from 46 to 26 (Figure 1b). 

**Figure 2:**
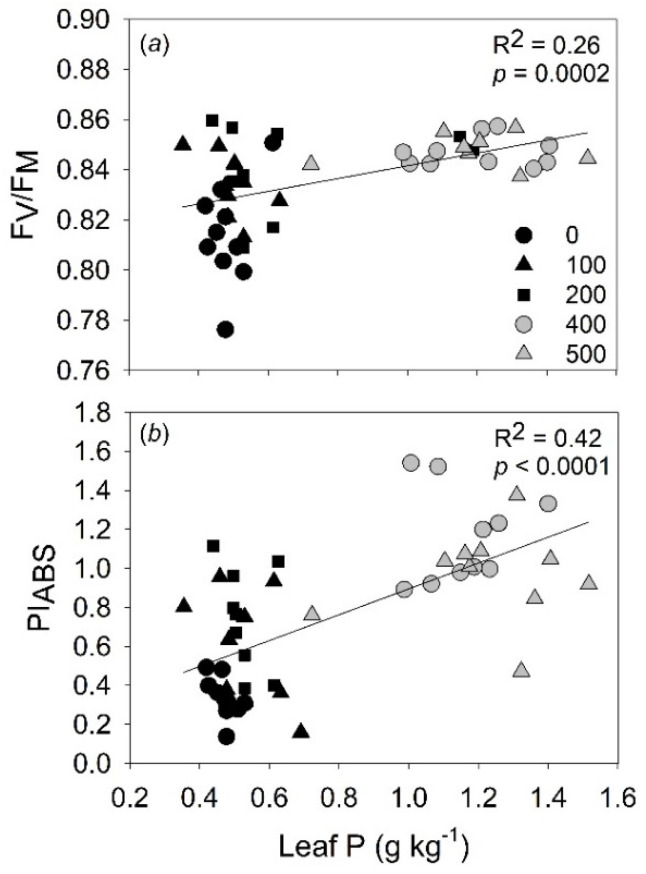
Influence of leaf phosphorus on photochemical efficiency of PSII (**a**) and performance index (**b**) of *Bertholletia excelsa* saplings in a P-deficient Amazonian Ferralsol.

The increase of P content in leaves favors the photochemical efficiency of PSII (F_V_/F_M_) and the performance index (PI_ABS_). Furthermore, F_V_/F_M_ and PI_ABS_ were correlated linearly with P content in leaves (Figure 2a,b).

**Figure 3:**
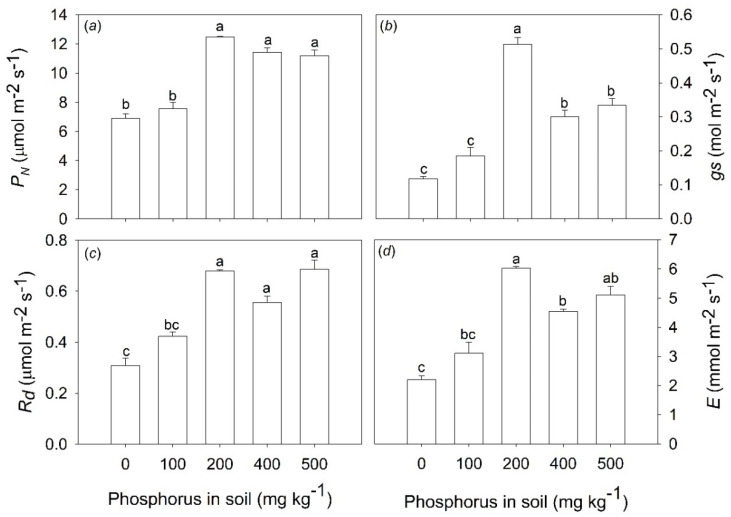
Net photosynthesis (**a**), stomatal conductance (**b**), dark respiration (**c**), and transpiration (**d**) of *Bertholletia excelsa* saplings under increasing doses of phosphorus in a P-deficient Amazon Ferralsol. Bars are the means and lines above the bars are the standard error. Different letters in the bars indicate significant differences between the means by the Tukey test at a 0.05 probability level.

The application of 200 mg P kg^−1^ in the soil leads to increased net photosynthesis (*P_N_*), dark respiration (*R_d_*), stomatal conductance (*gs*), and transpiration (*E*) (Figure 3a,d). The photosynthetic rates, for example, in the saplings subjected to 200 mg P kg^−1^ were approximately 50% greater when compared with plants under 0 and 100 mg P kg^−1^ in the soil (Figure 3a).

**Figure 4:**
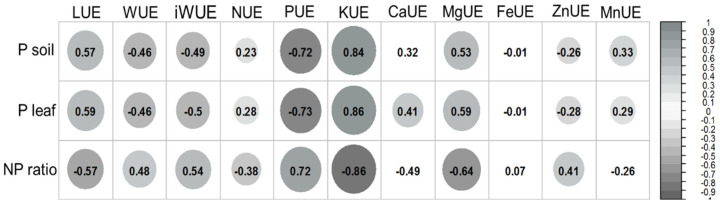
Correlation between phosphorus availability and resource use efficiency of *Bertholletia excelsa* saplings in a P-deficient Amazon Ferralsol. The significant correlations are represented by a gray circle, and the larger and darker the circle, the stronger the correlation. Abbreviations: LUE, light use efficiency; WUE, water use efficiency; iWUE, intrinsic water use efficiency; NUE, nitrogen use efficiency; PUE, P use efficiency; KUE, potassium use efficiency; CaUE, calcium use efficiency; MgUE, magnesium use efficiency; FeUE, iron use efficiency; ZnUE, zinc use efficiency; MnUE, manganese use efficiency.

The P content in leaves and soil were positively correlated with light use efficiency (LUE), nitrogen (NUE), potassium (KUE), magnesium (MgUE), and manganese (MnUE) use efficiency by the *B. excelsa* saplings (Figure 4). Calcium use efficiency (CaUE) was correlated with P contents in the leaf, but not with P soil (Figure 4). On the other hand, contents of P in leaves and soil were negatively correlated with water use efficiency (WUE), intrinsic water use efficiency (iWUE), P use efficiency (PUE), and zinc use efficiency (ZnUE) (Figure 4). No significant correlation was observed between the P contents in leaf or soil and iron use efficiency (FeUE) (Figure 4).

**Figure 5:**
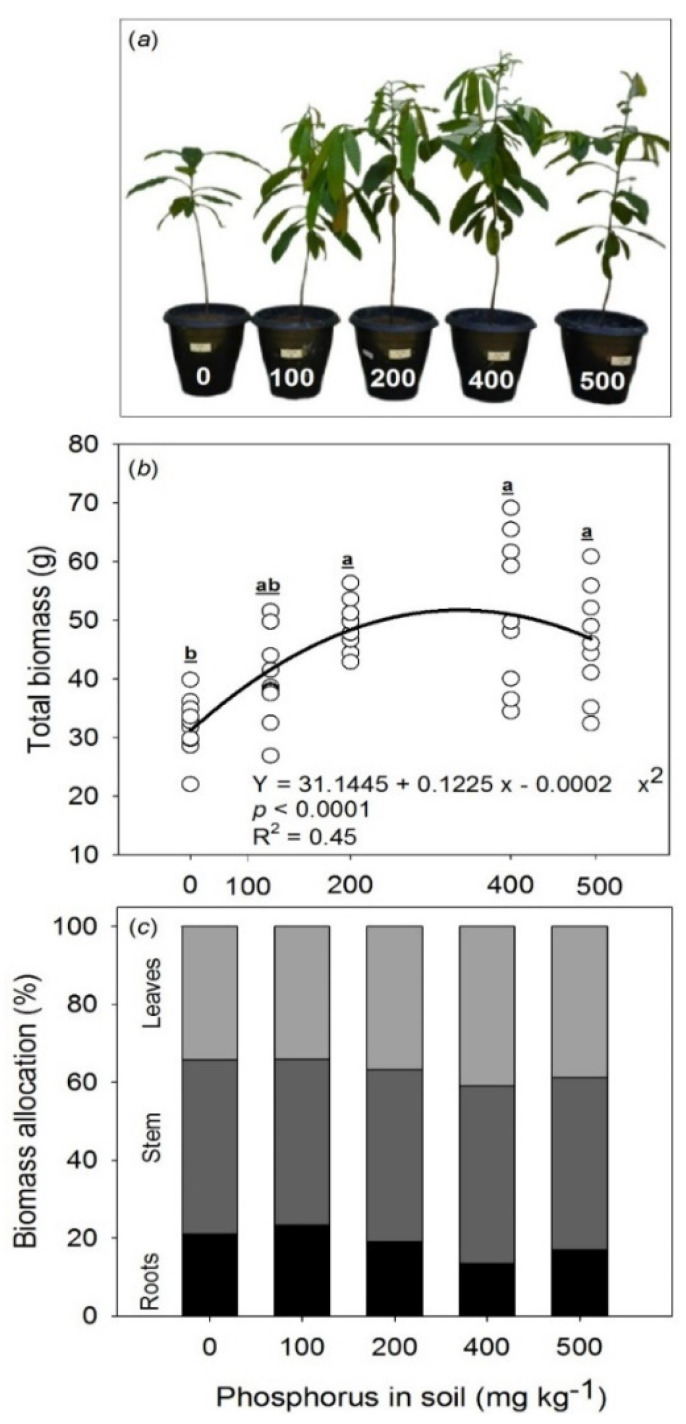
Influence of phosphorus soil availability on total dry biomass accumulation (**a**,**b**) and biomass allocation of *Bertholletia excelsa* with 24-month-old saplings (**c**) in P-deficient Amazon Ferralsol. Different letters indicate significant differences between the means by the Tukey test at a 0.05 probability level.

Phosphorus fertilization was shown to increase the total dry biomass of the saplings (Figure 5a,b). The increase in dry biomass values was observed from 200 mg P kg^−1^ added in soil (Figure 5b), with greater values in plants subjected to 400 mg P kg^−1^, which showed total dry biomass that was 66% greater than the control. The maximum point of the curve occurred at the coordinates x = 306.2 mg P kg^−1^ and y = 49.9 g (Figure 5b). In the control plants, about 21% of dry biomass was allocated to roots, while in plants under P only 16% of dry biomass was allocated to this organ (Figure 5c). In relation to shoot tissues (leaf + stem), control and fertilized plants allocated 79 and 86% of total dry biomass, respectively (Figure 5c).

Higher values of total dry biomass in plants under phosphorus fertilization were accompanied by higher growth rates based on plant diameter and height, leaf gain, and leaf area (Figure 5a). The stem diameter and height of the plants were 77% (under 400 mg P kg^−1^) and 44% (under 500 mg P kg^−1^) greater than those of the control plants (Table 1). Phosphorus fertilization also favored leaf area, which was increased by 94% in plants under 400 mg P kg^−1^, and leaf gain increased by 136% in plants under 200 mg P kg^−1^, when compared with the control plants, respectively (Table 1). Plants under P fertilization decreased the root/shoot ratio and there were no effects perceived on SLA (Table 1).

## 3. Discussion

Phosphate fertilization is a neglected practice in *B. excelsa* commercial plantations in the Central Amazon due, among other factors, to the fact that *B. excelsa* has high growth rates when compared to other native and exotic species, even in P-poor soils [16,17,18]. However, in this study, it was observed that the addition of P to soils enables *B. excelsa* to express its productive potential. Phosphorus fertilization increased dry biomass and also improved important physiological traits, such as electron transport (F_V_/F_M_ and P_ABS_), gas exchange parameters (*P_N_*, *gs*, *E*, and *R_d_*), light use efficiency (LUE) and nutrient (N, K, Ca and Mg) use efficiency in saplings of this species. These results suggest that P fertilization is a beneficial silvicultural practice for increasing growth rates in *B. excelsa* plantations, and corroborate with previous studies suggesting that P availability in the soil is a limiting factor for Amazon rainforest productivity [7,8,27]. Additionally, leaf P content was positively associated with fruit production by *B. excelsa* trees [13,26]. The P content of *B. excelsa* leaves without phosphate fertilization (0.42 g kg^−1^) found in this study is in accordance with what has been shown for *B. excelsa* plants. The leaf P levels varied from 0.1 to 1.2 g kg^−1^ for plants in different sites and plants of different ages, and the highest values of P leaf are related to the more fertile soils [11,12,13,21,22].

The values of the N:P ratio around 46 in the control treatment suggest that *B. excelsa* plants may be strongly limited by P. In the saplings fertilized with 400 and 500 mg P kg^−1^, the N:P leaf ratio reduction to 26 represents a significant physiological gain due to better N:P balance suiting leaf metabolism. However, despite of the substantial reduction of the N:P ratio of approximately 76%, *B. excelsa* saplings may still be limited by P, since the optimal value of the N:P ratio for most plants ranges from 10 to 20 [28]. Nevertheless, it is important to highlight that optimal N:P ratios depend on the species, growth rates, plant age, and plant parts [28].

Differences in P content and N:P values were found only at higher levels of P soil (400 and 500 mg P kg^−1^), but differences in chlorophyll *a* fluorescence, gas exchange parameters, and growth were observed at lower P levels (100 and 200 mg P kg^−1^). Thus, it is possible that even though there was no difference in the P content in the leaves of the treatments in which 100 and 200 mg P kg^−1^ were added to the soil, the increase in the availability of P in these treatments was sufficient to favor the physiological performance of *B. excelsa*. This can be visualized from the more sensitive measurements, such as photosynthesis-related parameters. The absence of changes in P content and N:P ratio in leaves in low P levels (100 and 200 mg P kg^−1^) is in agreement with the fact that nutrient contents of young and mature leaves are not good indicators of soil nutrient availability [29]. Other parts of the plants with old leaves and fine roots are the most appropriate indicators, and these responses are due to the fact that new and mature leaves have higher homeostatic regulation [29].

Phosphorus deficiency reduces the activity of the ATP synthase complex, causing lumen acidification, which increases heat energy loss. Furthermore, reduced ATP synthase activity might inhibit electron transport from plastoquinol (PQH_2_) to the cytochrome (Cyt) b6f complex that, in turn, prevents re-oxidation of plastoquinone. Consequently, it decreases the electron transport to the PSI, thus resulting in lower values of quantum yield (F_V_/F_M_) and performance index (PI_ABS_). This results in lower ATP production, which may reduce the production of NADPH. Thus, the Calvin–Benson cycle is limited by lower levels of ATP and NADPH, reducing CO_2_ assimilation rates [30,31,32]. It was observed that P fertilization enhanced photosynthetic rates (*P_N_*) of leaves from *B. excelsa* saplings under P-deficient conditions. Phosphorus availability in soil has been associated with increased *P_N_* rates, particularly in plants with the best photochemical performance [30,31]. In this study, the increase in P availability in the soil favored F_V_/F_M_ and PI_ABS_. Additionally, the enhancement of stomatal conductance (*gs*) was observed. With F_V_/F_M_ and PI_ABS_ being entirely complementary, to a certain extent, this support the hypothesis that greater *P_N_* in *B. excelsa* saplings fertilized with P can be explained by improving photochemical and gas exchange capacity.

The effects of P fertilization on *gs* are highly variable and depend on the species, site conditions, and availability of resources [33]. However, tropical tree species show a strong relation between Pi and *gs* (Pasquini et al., 2012), suggesting that stomatal function may be limited by the availability of P in these plants. Although the mechanisms by which P can influence *gs* have not been explored, the higher *gs* values have always been related to higher *P_N_* and, consequently, greater demand for CO_2_ [30]. Thus, in this study, the enhancement of *P_N_,* due to the addition of P to the soil, increased CO_2_ demand and this may have stimulated the increased *gs*. In addition, the greater *gs* resulted in increases in transpiration (*E*) and, consequently, a reduced WUE, which is represented by negative correlations between P leaf and WUE or iWUE. In this study, the reduction in WUE observed in *B. excelsa* saplings fertilized with P may indicate the absence of limitation by this resource, since the soil moisture was maintained in the field capacity condition. In contrast to WUE, the positive correlations demonstrate that the increased efficiency in the use of light (LUE) and nutrients (N, K, Ca, Mg, Fe, Mn) occurred in plants fertilized with P. This can be explained, in part, by the increase in photosynthesis rates and changes in the morphology of the plants (leaf area; root/shoot ratio), which may have favored both the efficiency in uptake and utilization of resources.

The greater efficiency in the use of nutrients is due to the plant’s ability to uptake and/or utilize nutrients and becomes more efficient when it produces more biomass with less absorbed nutrients [21,34]. In this study, the increase is due, especially, to the increase in *P_N_*, since changes in leaf nutrient contents were not observed between treatments and a reduction in root/shoot ratio was found, which represents, in part, a reduction in the capacity for nutrient uptake. Considering these data, it can be hypothesized that P fertilization in *B. excelsa* plants is extremely important for the more efficient use of the other available nutrients, and this should be considered an important silvicultural practice for the sustainability of commercial plantations of this species.

Several studies have shown a clear dependence between LUE and the CO_2_ assimilation rate [21,34]. In general, the increase in the leaf photosynthesis rate increases the LUE non-linearly, with the LUE reaching a maximum value at high photosynthesis rates [34]. Thus, the greater LUE values found in this study for saplings under P fertilization may be attributed, in part, to increases in the capacity of the utilization of light, which is acquired by improvements in photosynthesis. However, in addition to the increase in the capacity of use, the increase in the capacity to acquire light through the increase of the leaf area, leaf gain, and leaf biomass may also have contributed to the increase in LUE in *B. excelsa* saplings fertilized with P.

The increase of above-ground dry biomass, larger leaf area, and greater leaf gain contribute to the carbon gain since it is directly related to the photosynthetic capacity of the plants. Plants with larger leaf area and leaf gain have a larger surface area to capture light and, consequently, higher production of dry biomass. These parameters, and changes in the allocation of biomass, together with specific leaf area (SLA), are important above-ground indicators of plant strategies related to resource availability. While leaf area, leaf gain, and biomass allocation have often been used to measure site fertility [35], SLA is the most responsive to plant strategies related to light availability [36], which explains the lack of effects of P fertilization on SLA of *B. excelsa* saplings in this study.

Regarding dry biomass allocation, the decreasing root-to-shoot biomass ratio is a common response to P fertilization and occurs due to a complex group of adaptative responses, such as changes in gene expression levels, carbohydrate partition, root morpho-anatomical traits, and ethylene biosynthesis, that provided more carbon to the shoots, which resulted in increased shoot growth [37,38]. This can be verified by the greater increase in above-ground biomass, larger leaf area, and greater leaf gain, and this contributes to the carbon gain since it is directly related to the photosynthetic capacity of plants. Plants with larger leaf areas and leaf gains have larger surface areas to capture light and, consequently, a higher biomass content [39].

Dry biomass accumulation and growth rates in diameter and height are parameters that have already been applied to several tropical tree species in order to analyze and understand the balance between, and the levels of, nutrients in the soil and in the plant [23,39]. In this context, the plant responses almost always suggest that their physiological performance is limited by P [9,40]. This reinforces the hypothesis that *B. excelsa*, despite having high growth rates in relation to other native and exotic species, even in places with low availability of P, is limited by P. Thus, P fertilization in *B. excelsa* plantations can provide a considerable increase in production. Furthermore, the P content in the soil that is capable of inducing the maximum biomass production potential in young *B. excelsa* plants is about 300 mg P kg^−1^. Obviously, this value may vary with the physical characteristics of the forest environment, in particular, edaphic factors and microclimatic conditions (precipitation, light, and temperature).

## 4. Materials and Methods

### 4.1. Experiment Location and Plant Material

The experiment was conducted in a greenhouse with forced ventilation, located in the National Institute for Amazonian Research (INPA), Manaus, Brazil (3°05′30″ S; 59°59′36″ W). The temperature in the greenhouse varied from 28 °C at 8:00 a.m. to 34 °C at 12:00 a.m. and the values of photosynthetic photon flux density, measured at noon, were 1159 ± 6.13 μmol photons m^−2^ s^−1^. The *B. excelsa* saplings were produced by seminal propagation at Agropecuária Fazenda Aruanã S.A., located in the municipality of Itacoatiara, Amazonas state, Brazil (3°00′29″ S; 58°49′53″ W). The saplings used in this study were approximately 24 months old, 42 cm in height, 0.5 cm in diameter, and had four leaves. The experiment was carried out over six months.

### 4.2. Experiment Design

The treatments were as follows: the control (without phosphorus addition), and four treatments with the addition of 100, 200, 400, and 500 mg P kg^−1^ to the substrate used for growing the *B. excelsa* saplings. The substrate for cultivation of the saplings, in this study, was collected from the superficial layer (0–20 cm) of a very clayey, yellow Latosol, and, before applying P to the soil, this was subjected to air drying, sieving with a 2 mm mesh and then was used to fill 50 plastic pots of 10 L each. 

Soil samples from each of the pots were collected for chemical analysis, and the Ferralsol showed a pH of 4.2, the total nitrogen content was 0.72 g kg^−1^, the available phosphorus content was 1.0 mg kg^−1^ (P extracted with Mehlich-1), the calcium content was 0.05 cmolc kg^−1^ and magnesium content was 0.03 cmolc kg^−1^. From the soil chemical data, the necessity for liming [41] was estimated and compared it with another Ferralsol [42].

The liming applied to the substrate was a mixture of calcium carbonate (CaCO_3_) and magnesium carbonate (MgCO_3_) p.a in order to elevate the base saturation by 50%. This corresponds to 1.089 g kg^−1^ of calcium (Ca), and 0.26 g kg^−1^ of magnesium (Mg) per plastic pot of 10 L. After the application of the liming, the substrate was incubated for 20 days to react with the soil. During this period, soil moisture was maintained at around 30% (field capacity for latosols in relation to dry soil weight) by means of irrigations with distilled water, and moisture levels were monitored by weighing the pots every 4 days [43]. 

After 20 days of liming, the pH of the soil increased from 4.1 to 5.6, and, subsequently, the substrate was submitted to air-drying for fertilization. Thus, 4.00 g kg^−1^ of nitrogen (N), 0.631 g kg^−1^ of potassium (K), 0.26 g kg^−1^ of sulfur (S), 0.80 mg kg^−1^ of boron (B), 0.50 mg kg^−1^ of zinc (Zn), 1.50 mg kg^−1^ of copper (Cu), 0.15 mg kg^−1^ of molybdenum (Mo) and 3.60 mg kg^−1^ of manganese (Mn) were applied to the substrate of each pot. Phosphorus fertilization was performed at the same time. After the application of the macro- and micronutrients, the soil was incubated for 20 days.

With the exception of sulfur, which was applied in solid form, all macro- and micronutrients were applied in a dissolved form. Nitrogen was reapplied in installments every 40 days, using urea [CO(NH_2_)_2_] as the source, in a total dose of 400 mg kg^−1^, and was divided into four individual applications of 100 mg kg^−1^. The supply of P and the standard fertilization were made with the use of salt of phosphorus (KH_2_PO_4_ and (NH_4_) H_2_PO_4_), which was balanced so that only the P contents were increased in all treatments of phosphate fertilization. 

After 20 days of soil incubation with the nutrient solution, the transplantation of the *B. excelsa* saplings to their pots containing the substrate was performed. The saplings were carefully removed from the old container, transferred to a basin with distilled water to facilitate the complete transfer of the root system and then planted in the new substrate. The saplings were arranged on benches suspended in a greenhouse and subjected to periodic rotation to avoid possible effects of positioning within the greenhouse. All data were collected 182 days after the transplanting of the saplings.

### 4.3. Plant Growth and Morphological Traits

Growth measurements (height, diameter, leaf area, and the number of leaves) were performed at 21-day intervals throughout the all-experimental period (six months) and totaled nine measurements. The height of the saplings (considered up to the apical bud) was measured using a tape graduated in centimeters. The diameter was measured at the base of the plant close to the ground using a digital caliper (Mitutoyo, CD-8 CX-B). Leaf area (LA) was measured using a leaf area meter (CID Inc., Camas, WA, USA). For the determination of the mass, the plants were sectioned in shoot and root tissues and later conditioned in a forced ventilation oven at 70 °C, until reaching a constant mass. The relative growth rates in height (RGRH), diameter (RGRD), leaf gain, and shoot/root ratio were calculated [44]. Specific leaf area (SLA) was calculated using the ratio between leaf mass, which was dried at 70 °C to a constant weight, and the leaf area from leaf discs of a known area [36].

### 4.4. Leaf Nutrient Content

Two healthy and fully expanded leaves were taken from saplings at the end of six months of experimentation. The leaf samples were dried in an oven at 65 °C for constant mass evaluation. The total N was determined using the Kjeldahl method. Macronutrients (Ca, Mg, P and K) were extracted with a 3:1 nitric-perchloric solution, and the contents of these nutrients were determined via atomic absorption spectrometry (Perkin-Elmer 1100B, Uberlingen, Germany) and P was determined by spectrophotometry at 725 nm [45]. The P limitation was determined according to the N:P ratio [28].

### 4.5. Leaf Gas Exchange and Resource Use Efficiency

Net photosynthesis per unit area (*P_N_*), stomatal conductance (*gs*), transpiration (*E*), and dark respiration (*R_d_*) were measured using a portable infrared gas analyzer (Li-6400; Li-Cor, Lincoln, NE, USA) with a leaf chamber area of 6.0 cm^2^ and light provided by the red and blue LEDs (665 and 470 nm, respectively). The measurements were made at the end of six months of experimentation, between 8:00 a.m. and 12:00 p.m. on healthy, fully expanded, and mature leaves. The airflow through the chamber was approximately 400 µmol s^−1^, the CO_2_ concentration [CO_2_] of the flow rate into the chamber was 400 ± 1 µmol mol^−1^, the block temperature was 31 ± 1 °C, and H_2_O vapor was about 21 ± 1 mmol mol^−1^ [21]. 

Instantaneous water use efficiency (WUE) was calculated as *P_N_*/*E*, water intrinsic use efficiency (WIUE) was calculated as *P_N_*/*gs*, nutrient use efficiency was calculated as *P_N_*/nutrient and light use efficiency (LUE) or apparent quantum yield (α) was obtained from adjusting photosynthetic photon flow density (PPFD) responses in *P_N_*-PPFDi curves. These curves were obtained for a PPFD between 0 and 2000 µmol photon m^−2^ s^−1^ in 11 steps (2000, 1500, 1000, 750, 500, 250, 100, 75, 50, 25, and 0 µmol photon m^−2^ s^−1^) in decreasing order and were taken on the same leaf. An exponential model was used for adjusting the PPFD response in *P_N_* area-PPFDi curves [21] for each sapling according to the following Equation (1).

(1) $$\;P_{N}=\left(P_{N\;max\;area}+R_{d}\right)\left\{1-exp\left[\frac{-\alpha I}{P_{N\;max\;area}+R_{d}}\right]\right\}-R_{d\;}$$

where *I* is the irradiance (PPFD), *P_N_* is the net photosynthesis per unit area (µmol m^−2^ s^−1^), *P_N_*_max area_ is the maximum net photosynthesis per unit area, *R_d_* is the dark respiration (µmol m^−2^ s^−1^) corresponding at the *P_N_* area value when *I* = 0 µmol m^−2^ s^−1^, and *α* is the apparent quantum yield (mol CO_2_ mol quanta^−1^). *P_N_*-PPFDi curves were adjusted using the Levemberg-Marquardt algorithm in Statistica 7.0 software (StatSoft, Inc., Tulsa, OK, USA) to estimate *P_N_* max area.

### 4.6. Chlorophyll a Fluorescence

The determination of chlorophyll *a* fluorescence was performed on the same leaves used to determine the gas exchange and was conducted using a plant efficiency analyzer (PEA, MK2-9600, Hansatech, Norfolk, UK). The measurements were made between 8:00 a.m. and 12:00 p.m. on healthy and fully expanded mature leaves [22]. The leaves were subjected to a 30-min period of adaptation to darkness. Then, the leaf was exposed to a 1-s excitation pulse of high irradiance (3000 μmol photon m^−2^ s^−1^) with a wavelength of 650 nm [12]. The parameters related to polyphasic chlorophyll a fluorescence transient were obtained from specific software (Handy PEA software—v 1.3). The maximum yield of photochemistry (F_V_/F_M_) and PI_ABS_ were calculated according to the JIP-test [22].

### 4.7. Data Analysis

The experimental design was completely randomized with five treatments (control—without fertilization, 100, 200, 400, and 500 mg P kg^−1^ addition) and ten replicates (saplings). The data were preliminarily verified for the assumptions of normality and homogeneity of variance by Shapiro-Wilk and F tests, respectively. Then, the effects of the level of phosphorus on *B. excelsa* saplings were evaluated using one-way ANOVA, and means were compared by the Tukey test at a 0.05 probability level. Regression analysis was used for explaining the effects of the P content in leaves and physiological variables. Statistical analysis was performed using statistical software and R programming language.

## 5. Conclusions

Phosphorus fertilization increased the growth of *B. excelsa* saplings due to improved photochemical efficiency, gas exchange, nutrient use efficiency, and changes in biomass allocation. Thus, P fertilization is recommended for improving forest plantations of *B. excelsa* saplings and the level of 306.2 mg P Kg^−1^ in the soil was needed to obtain the maximum yield. 

## Figures and Tables

**Table 1:** Influence of soil phosphorus availability on growth and morphological traits of *Bertholletia excelsa* saplings. The relative growth rates in height (RGRH), diameter (RGRD), and specific leaf area (SLA). Different letters on the same line indicate significant differences between the means by the Tukey test at a 0.05 probability level.

Traits	Phosphorus Addition in Soil (mg kg^−1^)				
	0	100	200	400	600
RGRH (cm day^−1^)	4.8 ± 0.4 C	5.0 ± 0.5 BC	6.8 ± 0.31 AB	7.9 ± 0.6 A	7.6 ± 0.6 A
RGRD (mm day^−1^)	0.6 ± 0.1 BC	0.7 ± 0.1 ABC	0.9 ± 0.04 AB	0.9 ± 0.1 A	0.9 ± 0.1 AB
Leaf area _total_ (cm^2^)	1.6 ± 0.1 B	2.1 ± 0.1 B	2.8 ± 0.23 AB	3.2 ± 0.3 A	3.0 ± 0.2 AB
SLA (cm g^−1^)	163 ± 4.2 A	164 ± 4.5 A	169 ± 4.02 A	163 ± 3.0 A	166 ± 6.0 A
Leaf gain (%)	272 ± 1. C	415 ± 0.9 BC	642 ± 1.32 A	610 ± 1.4 A	567 ± 1.1 AB
Root/Shoot	0.3 ± 0.03 A	0.3 ± 0.03 A	0.3 ± 0.03 AB	0.2 ± 0.02 B	0.2 ± 0.02 B

## Data Availability

Not applicable here.
